# Interaural Level Differences Do Not Suffice for Restoring Spatial Release from Masking in Simulated Cochlear Implant Listening

**DOI:** 10.1371/journal.pone.0045296

**Published:** 2012-09-20

**Authors:** Antje Ihlefeld, Ruth Y. Litovsky

**Affiliations:** 1 University of Wisconsin Waisman Center, Madison, Wisconsin, United States of America; 2 New York University, Center for Neural Science, New York, New York, United States of America; University of Nevada Las Vegas, United States of America

## Abstract

Spatial release from masking refers to a benefit for speech understanding. It occurs when a target talker and a masker talker are spatially separated. In those cases, speech intelligibility for target speech is typically higher than when both talkers are at the same location. In cochlear implant listeners, spatial release from masking is much reduced or absent compared with normal hearing listeners. Perhaps this reduced spatial release occurs because cochlear implant listeners cannot effectively attend to spatial cues. Three experiments examined factors that may interfere with deploying spatial attention to a target talker masked by another talker. To simulate cochlear implant listening, stimuli were vocoded with two unique features. First, we used 50-Hz low-pass filtered speech envelopes and noise carriers, strongly reducing the possibility of temporal pitch cues; second, co-modulation was imposed on target and masker utterances to enhance perceptual fusion between the two sources. Stimuli were presented over headphones. Experiments 1 and 2 presented high-fidelity spatial cues with unprocessed and vocoded speech. Experiment 3 maintained faithful long-term average interaural level differences but presented scrambled interaural time differences with vocoded speech. Results show a robust spatial release from masking in Experiments 1 and 2, and a greatly reduced spatial release in Experiment 3. Faithful long-term average interaural level differences were insufficient for producing spatial release from masking. This suggests that appropriate interaural time differences are necessary for restoring spatial release from masking, at least for a situation where there are few viable alternative segregation cues.

## Introduction

In natural acoustic settings, source segregation can be difficult, resulting in deteriorated speech intelligibility even for normal hearing listeners. The problem is aggravated for cochlear implant listeners who often struggle to understand a target source under these conditions. For normal hearing listeners, spatial differences between competing sources can provide potent cues for directing attention to a target. Specifically, when a target and a masker source are spatially separated, it is usually easier to understand the target than when both sources originate from the same direction, a phenomenon known as spatial release from masking (SRM; e.g., see [1], [2]). For a fixed performance threshold, SRM can be expressed as the difference in target-to-masker broadband energy ratio (TMR) between these two spatial configurations.

Three factors typically contribute to SRM: the acoustic head shadow at the better ear of the listener (up to 11 dB), binaural decorrelation processing (2–3 dB), and spatial attention (up to 15 dB; see, e.g., [3], [4], [5], [6]). When a target is spatially separated from a masker, the acoustic head shadow typically leads to higher TMR in one ear over the other, referred to as the better ear. For instance, when the target is to the left, and masker in front, the TMR in the left ear is higher than the TMR in the right ear. In addition to this monaural benefit, binaural decorrelation processing can combat energetic masking by allowing a listener to detect a target when the interaural time differences (ITDs) between target and masker differ (e.g., [3]). Both better ear effect and binaural decorrelation effect vary directly with the acoustic fidelity and amount of binaural differences. For attention-driven SRM to occur, however, at least for normal hearing listeners, spatial separation between competing sources and resulting binaural differences do not need to be large, provided that listeners can perceive the sounds at spatially distinct locations and attend to the direction of the target source ([7], [8], [9], [10]).

Bilateral cochlear implants are provided to a growing population of patients who are deaf, with the aim of improving their ability to use spatial cues for sound localization and source segregation (e.g., [11], [12]). Clinical cochlear implant processors can convey spatial information via interaural level differences (ILDs) through the slowly varying portions, or envelopes, of the sound reaching the ears ([11], [13]), but generally fail to transmit ITDs (e.g., see [14], [15]). Because, at least in the long-term across-time average, cochlear implant processors can preserve ILDs, this allows cochlear implant listeners to benefit from acoustic head shadow ([12], [16]). Moreover, ILDs can enable them to hear a sound source from a distinct spatial location ([17], [18]). This should allow cochlear implant listeners to deploy spatial auditory attention to segregate a target sound from the acoustic mixture and selectively listen to it. Spatial attention could be especially helpful to cochlear implant listeners, who typically cannot utilize alternative segregation cues, such as pitch and onset differences ([19], [20]). Moreover, at least for normal hearing listeners, previous work demonstrates that ILDs can be powerful cues for segregating concurrent synthetic vowels ([21]).

Surprisingly, previous work with cochlear implant listeners shows little if any release from masking due to spatial attention. In a representative study with normal hearing listeners, SRM was found to be larger for speech embedded in speech than for speech in noise that was spectrally matched to that of the speech (5.7 dB for speech and 4.5 dB for noise masker, [22]). This finding is consistent with other previous work showing that normal hearing listeners could utilize spatial attention when the targets were perceptually similar to each other (e.g., speech embedded in speech from a same-sex talker), but that spatial attention generally does not help performance much for conditions where target and masker were sufficiently perceptually different (e.g., speech embedded in noise, or speech embedded in speech from a different-sex talker; [23], [4], [5]). However, when bilateral cochlear implant users were tested on similar stimulus conditions as the normal hearing listeners, a different trend in SRMs emerged, with 2.2 dB release for speech and 4.2 dB or noise maskers ([24]). SRM in cochlear implant listeners was smaller for speech in speech than for speech in noise. This was observed despite preserving both ILDs and across-electrode onset ITDs.

Together, these findings indicate that high-fidelity, or “clean,” long-term ILDs alone do not suffice for directing spatial attention when short-term ITDs and ILDs fluctuate dynamically and that listeners also need access to faithfully encoded ITD cues ([22],[24]). Alternatively, spatial information may be available at the periphery of the auditory system, but perhaps as a result of auditory deprivation, cochlear implant listeners may sub-optimally pool that information ([25]).

Through vocoding, here we simulated aspects of cochlear implant listening to examine what factors may prevent listeners from successfully deploying spatial attention to a target source. In general, vocoding techniques offer insights into cochlear implant listening by providing acoustic cues that are degraded approximately similarly to the types of speech cues that a cochlear implant processor may provide ([26]). Typically, for NH listeners identifying four- to eight-channel vocoded speech in quiet, intelligibility scores are similar to those obtained from cochlear implant listeners ([27], [28]). The current study used stimuli generated with a novel processing strategy, “chess-vocoded” speech, to simulate cochlear implant listening and manipulate fidelity of ITD cues. Traditional noise vocoded speech was filtered into non-overlapping frequency bands and temporally gated on and off using 100 Hz window functions. This resulted in 10 ms long and 1/3 octave wide glimpses that were regularly structured like on-off fields akin to the fields in a chess game. Each glimpse in isolation was an unintelligible burst of narrowband noise. However, when bound together appropriately across time and frequency, the “chess glimpses” formed intelligible speech, as verified by testing speech identification in quiet.

Presumably because the temporal envelope was low-pass filtered at 50 Hz and because temporal fine structure information was replaced with noise, these vocoded stimuli did not elicit a strong temporal pitch. Moreover, when presented in a two-source mixture, the synchronous gating of stimulus energy introduced comodulation cues that encouraged perceptual fusion of glimpses across different sources. Together, reduced pitch cues and misleading comodulation simulated a situation where the listener struggles to segregate target from masker, much like one may expect the perceptual experience of a cochlear implant listener to be in a crowded acoustic setting.

We tested the hypothesis that ITD fidelity of the target sound is necessary to improve the ability to spatially direct attention, even when long-term ILD cues robustly encode directional sound. As a control, Experiment 1 measured SRM in unprocessed speech with full spatial cues. Experiments 2 and 3 measured speech intelligibility for spatially co-located and separated sources for vocoded speech. In Experiment 2, ITD and ILD cues faithfully encoded source directions. ITDs and ILDs in these “clean” conditions were very similar in magnitude to binaural differences in unprocessed speech with full spatial cues in Experiment 1. In Experiment 3, ITDs were scrambled, while maintaining the long-term average ILDs. Based on our hypothesis, we expected listeners to receive less spatial attention benefits when ITD cues were scrambled than when they cleanly encoded the source directions.

**Figure 1:**
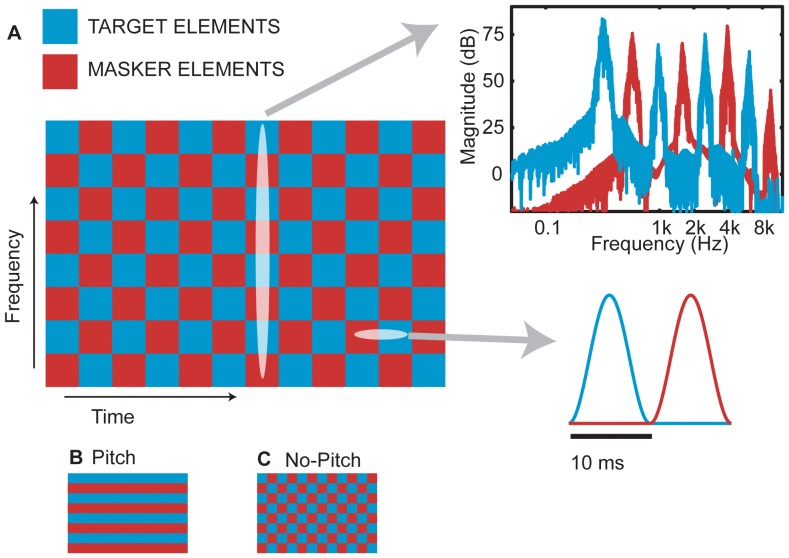
Illustration of the two vocoding strategies. A) Magnitude of the filters for generating a mixture of one target and one masker source in *No-Pitch* configuration. Time-frequency “chess” glimpses are shown in blue for the target and in red for the masker. The upper right insert shows example target and masker spectra, when the two sources were processed with this filter, averaged over a 10 ms time period (cf., vertical ellipse in 1.A). Target and masker spectra generally have little mutual energetic overlap (compare peak energy levels of each source at 75 dB to intersection points between blue and red curves at 25 dB, a dramatic 50 dB difference). The lower insert illustrates the shape of the time windowing that was applied over each 20 ms time period (cf., horizontal ellipse in 1A). B) and C) Side-by-side comparison of filters for a target-masker mixture in *Pitch*, and *No-Pitch* configurations. In both configurations, target and masker glimpses occupy mutually exclusive parts of the spectrum. Furthermore, because of the time windowing, in both *Pitch* and in *No-Pitch* configuration, all target and masker glimpses within each 10-ms time slice are co-modulated with each other, enhancing perceptual fusion between all elements in the mixture. In *Pitch* configuration, each source only occupies fields in half of the spectral channels (resulting in co-modulated glimpses with place pitch, i.e., a stripy filter pattern). In *No-Pitch* configuration, glimpses from both sources can occupy all spectral channels, constrained so that within each 10-ms time slice each source only occupies half of the channels (resulting in co-modulated glimpses without place pitch, i.e., a chess board filter pattern).

In both Experiments 2 and 3, target and masker glimpses did not overlap in time and frequency, so that the TMR was theoretically infinite in the target frequency bands (see Fig. 1 for an illustration of the spectrogram of the source mixture). Therefore, in both cases, if the listener knew which glimpses to attend to, the target should have been intelligible. Moreover, this stimulus design caused little mutual energetic masking between the two competing sources, strongly limiting the possibility that better ear acoustic advantage and binaural decorrelation processing could generate SRM.

We also manipulated place pitch cues for selecting the target from the mixture. In both Experiments 2 and 3, allocation of target energy could be either limited to only the odd or only the even frequency bands. Despite much reduced or absent temporal pitch cues from the vocoded speech envelopes, this frequency allocation scheme could provide a place pitch cue to segregate the target from the masker (“*Pitch*” conditions). Alternatively, target glimpses could fall across the entire spectrum of the sound mixture, such that target and masker place pitch heights were weak and highly similar (“No-*Pitch*” conditions). In the *Pitch* conditions, place pitch and spatial cues both allowed the listener to selectively attend to the target, whereas in the *No*-*Pitch* conditions listeners had to rely on spatial cues to direct their attention. Therefore, we expected SRM to be smaller in the *Pitch* than in the *No*-*Pitch* conditions.

## Materials and Methods

### Experiment 1– Full Speech, Full Spatial Cues

#### Stimulus generation

Raw speech stimuli were taken from a closed set corpus ([29]). The words were monosyllabic and not co-articulated, uttered by four female talkers. The target phrase started with the words “*Take the*…,” followed by an adjective and a noun. The adjectives were *big, dark, green, new, old, red, small*; the nouns were *chairs, desks, gloves, pens, shoes, socks, toys*. The masker phrase started with silence during the target words “*Take the*…,” and consisted of only an adjective and a noun. Both target and masker adjectives and nouns were taken from seven possible choices, constrained to differ from each other in each trial. All words within a target phrase or masker phrase were uttered by one talker. However, talkers of the target and masker sentence differed.

Stimuli were normalized in root mean square (RMS) and processed with HRTFs measured on a Knowles Electric Mannequin for Acoustic Research (KEMAR). HRTFs were anechoic, for a source at 0 degree and at 90 degree to the left of the listener. HRTFs were normalized so that the RMS in the left ear was the same for both sets. All processing was implemented in MATLAB 2009a.

The masker level was fixed at 70 dB SPL; the target level was set to 26, 38, 50, 62, 70, and 82 dB SPL. The binaural signals for the two sources were summed to produce the stimulus, resulting in target to masker ratio (TMR) in terms of broadband energy levels of −44, −32, −20, −8, 0, and 12 dB.

The masker was always presented from the 0 degree location; the target from 0 degree (co-located) or from 90 degrees to the left (spatially separated). Spatial configuration was blocked such that within each block of 48 trials the target location was held fixed. Target location alternated across blocks, counter-balanced across listeners.

#### Testing procedures

Stimuli were digital-to-analog converted, amplified using Tucker Davis System 3 hardware and presented over Sennheiser HD 580 headphones to the listener while seated in a sound-attenuated IAC chamber. After each trial, response buttons appeared on a graphical user interface, listing seven possible adjectives and seven possible nouns; the listener responded with a button press. Listeners were instructed to attentively listen to the voice saying “Take the,” focusing on voice qualities and spatial location, and to report the keywords that were spoken by the same talker. After each response, feedback was provided through display of the correct answer on the screen.

Listeners completed the experiment in one session, consisting of 18 blocks of 48 trials each, resulting in 72 trials per TMR and spatial configuration.

#### Listeners

Eighteen different listeners, all normal-hearing native speakers of American English (ages 18–22) participated in the study, six in each of the three experiments. Listeners were paid to partake in the study. Prior to testing all listeners passed a hearing test. Hearing thresholds were within normal range and spectrally symmetrical across the two ears with no more than 5 dB difference between left and right ear thresholds at each tested frequency (250, 500, 1000, 2000, 4000, 6000, 8000 Hz). This study was approved by the University of Wisconsin-Madison Health Sciences Institutional Review Board. All participants in this study gave written informed consent as approved by the University of Wisconsin-Madison Health Sciences Institutional Review Board.

#### Data analysis

For each spatial configuration in each experiment, we first calculated the logodds-ratio, i.e., the difference between logit-transformed psychometric functions for spatially separated and co-located configurations (cf., [30], [31]). We were interested in comparing SRM across different experimental conditions at a fixed 0 dB TMR, even when overall performance and variability of the underlying decision variables differed across these conditions. To that end, the resulting logodds-ratios were then transformed into z-scores, resulting in SRMs that were normalized in units of standard deviation. For each subject, masking condition and TMR, the logit-transform of probability correct score p was defined as logit(p)  =  ln(p/(1−p)). Probability values were thresholded such that for p smaller than chance performance (1/7), p was set to equal 1/7; for p greater than 1−1/N, where N is the number of trials, p was set to equal 1−1/N.

The logodds-ratio R was defined as the difference between probability correct scores in spatially separated versus co-located configurations, where
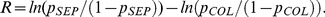
(1)


The variance of R equaled
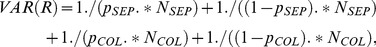
(2)where N_SEP_ and N_COL_ equaled the number of trials in the separated and co-located configurations, respectively. Note that throughout this study, N_SEP_ equaled N_COL_. SRM was defined as the z-score of the logodds-ratio between spatially separated and co-located performance, calculated as



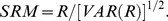
(3)SRM was calculated by subtracting logit-transformed percent correct scores obtained in the spatially separated conditions from those obtained in the co-located conditions at 0 dB TMR, normalized to z-units. Similar to what a d-prime analysis of perceptual sensitivity can achieve, this z-unit transform normalizes the observed mean difference between two random variables by the estimated standard deviation of that difference. Calculated this way, a hypothetical 10% SRM for co-located performance at 50% correct, spatially separated performance at 60% correct, and 72 trials per condition, translates to 1.2 z-units; whereas a 10% SRM for co-located performance at 85% correct translates to 1.9 z-units.

Data were analyzed with repeated measures analysis of variance (ANOVA) using the open-source package CLEAVE (T.J. Herron). For completeness, we report two measures of effect size: the estimated partial effect size for which a factor accounted after excluding all other factors in the design, partial eta square, η_p_^2^, and partial omega square, ω_p_^2^, which differs from η_p_^2^ in that it is adjusted for group mean squared error ([32]).

#### Acoustic properties of the stimuli

A binaural cue analysis quantified the fidelity of spatial information in the stimuli (see methods in [33]). For each frequency band and utterance, ITDs were estimated by cross-correlating left- and right-ear vocoded speech for interaural delays ranging from −900 to 900 µs. These cross correlation functions were then normalized by the square root of the product of the squared left and right ear signals to yield the normalized interaural correlation function. The interaural delay of the peak in the normalized cross correlation function was used to estimate the ITD in the stimulus. ILDs were calculated by subtracting the left ear RMS from the right ear RMS.

### Experiment 2– Clean ITDs and ILDs

#### Stimulus generation

Speech materials were identical to those in Experiment 1. Utterances were processed to produce intelligible signals simulating aspects of cochlear implant listening (e.g., [26]). Resulting signals were composed of narrowband-noise-burst elements that were by themselves unintelligible. A related approach had previously been used for pointillistic speech with tonal elements ([34]). Here, all utterances were normalized in RMS, and band-pass filtered into eight fixed frequency bands of 1/3 octave width, with center frequencies spaced between 300 Hz and 10 kHz evenly along the cochlea according to Greenwood’s formula ([35]). Band-pass filters simulated cochlear implant stimulation with 4 dB/octave frequency roll-off ([36]). The envelope of each band was extracted using the Hilbert transform and low-pass filtered with 50 Hz.

Envelopes were then pulsed on and off with a 50 Hz window function. A cycle of the window function comprised a 10 ms Tukey window with 5 ms rise and fall time (i.e., 50% duty cycle) and 10 ms of silence. Stimuli were generated both with 0 and with pi starting phase of the window function. For each utterance and frequency band, a random low-noise noise token was generated with bandwidth and center frequency corresponding to that of the frequency band of the envelope ([37]). Low-noise noise was chosen to minimize possible random degradation of the speech envelope introduced by amplitude fluctuations in the noise carrier. The temporally gated envelopes were each multiplied with their corresponding random low-noise noise token. Stimuli were then band-pass filtered with the same 1/3 octave filter as before and processed with the same HRTFs as in Experiment 1, generating left and right ear signals. Finally all eight bands of the utterance were added, producing noise-vocoded speech with high-fidelity ITD and ILD cues in both carriers and envelopes.

Two different types of acoustic mixtures were generated (cf., Fig. 1). In the continuous spectral band condition (*“Pitch”*), vocoded speech components with 0-phase- and pi-phase-envelope functions were added, subject to the constraint that target and masker signals were spectrally non-overlapping. One half of the frequency bands were assigned to the target and the other half to the masker, such that one source always used all odd bands, and the other source used all even bands (Fig. 1B). In the discontinuous spectral condition (*“No-Pitch”*), both the target and masker comprised all eight-frequency bands, subject to the constraint that the envelope starting phases within each frequency band were phase-shifted by pi between the two sources, and the envelope starting phases across frequency bands were phase-shifted by pi within each source (Fig. 1C). The resulting spectrograms in the continuous *Pitch* case consisted of frequency “slices” for the two sources; in the discontinuous *No-Pitch* case they consisted of “dice” akin to a chess-board pattern.

Here, we were interested in SRM obtained under conditions in which level cues typically do not help listeners perform the task. Therefore, only a −3 dB to +3 dB range of TMRs was tested in Experiments 2 and 3. Previous work shows that level cues are largely ineffective over such a small range ([23], [38], [5]).

**Figure 2:**
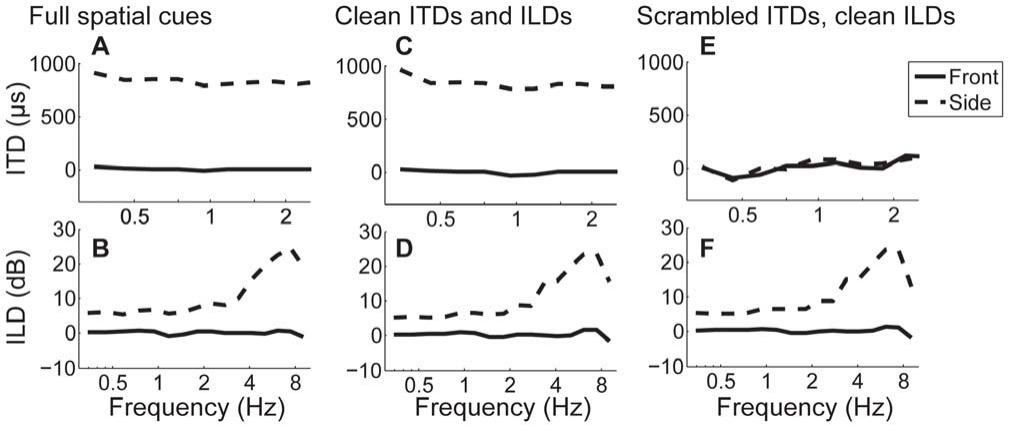
Acoustic properties of spatial cues. Across-time average interaural time differences (ITDs, top row, A, C, E) and interaural level differences (ILDs, bottom row, B, D, F) as a function of frequency. A, B) Unprocessed speech with high-fidelity spatial cues, from Experiment 1. ITDs and ILDs are zero in the Front configuration and considerably greater than zero in the Side configuration (in each panel, dashed lines are above solid lines). C, D) Chess-vocoded speech with high-fidelity ITDs and ILDs, from Experiment 2. ITDs and ILDs are approximately similar to those in the unprocessed condition (compare panels A and C, and B and D). E, F) Chess-vocoded speech with scrambled ITDs and high-fidelity ILDs, from Experiment 3. ITDs are close to zero in both Front and Side configuration (ITD pattern differs from those in panels A and C). ILDs, however, are approximately similar to those in the unprocessed and clean-ITD conditions.

Figures 2C and D show the long-term ITD and ILD cues, averaged across all utterances in the corpus when the utterance was processed with Front or Side HRTFs (denoted by solid or dashed lines). Similar to the full speech condition in Experiment 1 (cf., Figs. 2A and B), across-time average ITDs are close to 0 us in the Front and close to 800 µs in the Side conditions. ILDs are close to zero throughout the range of frequencies for the Front location, and increase with increasing frequency from 5 dB up to approximately 25 dB in the Side location.

#### Testing procedures

Procedures were similar to those in Experiment 1, except that prior to Experiment 2, listeners were screened with a brief ten-trial three alternative forced choice (3 AFC) spatial sensitization task to ensure that they could identify the direction of vocoded speech. Stimuli were presented from the left, right, or center (note that unlike the left and center stimuli, right stimuli were not used during the remainder of the experiment). Listeners were asked to report whether they heard the sound as coming from left, right or center. All listeners were able to perform this task with 100% response accuracy.

After the spatial sensitization task and at the beginning of each test session, listeners were tested on speech identification in quiet to ensure that they could identify the vocoded speech. This test consisted of two blocks of 48 trials each.

Listeners completed testing on 18 blocks of vocoded speech identification with a competing talker and were encouraged to guess if they were unsure about what they heard. Within a block of 48 trials, the spatial configuration between the competing sources was held fixed. TMR and spectral cue varied randomly from trial to trial such that each combination was presented once before all of them were repeated. During the two sessions, subjects performed 72 trials in each stimulus condition.

### Experiment 3– Scrambled ITDs and Clean ILDs

#### Stimulus generation

Stimuli were identical to those in Experiment 2, except for two differences. Temporal gating was only applied after HRTF processing. Within a frequency band, left and right ear carriers consisted of independent tokens of low-noise noise. When played from Front or Side direction, listeners could hear the stimuli as originating from the front or from the side, albeit with greater perceived spatial width.

Figures 2E and F show long-term ITD and ILD cues, averaged across all utterances in the corpus. Similar to acoustic cues in Experiments 1 and 2, here, ILDs in the Front condition equal 0 dB and in the Side condition range from 5 dB to 25 dB. Moreover, long-term across-time averaged ITDs are close to 0 in the Front condition. Unlike in Experiments 1 and 2, however, as a direct result of the modified stimulus generation, ITDs in the Side condition are also close to 0. Therefore, here, one could not select the target from the mixture based on ITD cues. Instead, listeners needed to focus on ILDs to select the target message.

#### Testing procedures

Procedures were similar to those in Experiment 2.

## Results

### Experiment 1

Figures 2A and B show ITD and ILD acoustic spatial cues for the stimuli in Experiment 1. Cues for the source location in front and to the side are plotted with solid and dashed lines, respectively. Acoustic spatial cues differed distinctly between the two locations: Across the entire sound spectrum, ITDs and ILDs in the front condition were close to 0, whereas in the side condition, ITDs equaled around 800 µs and ILDs increased with increasing frequency, covering a range between 5 and 25 dB.

**Figure 3:**
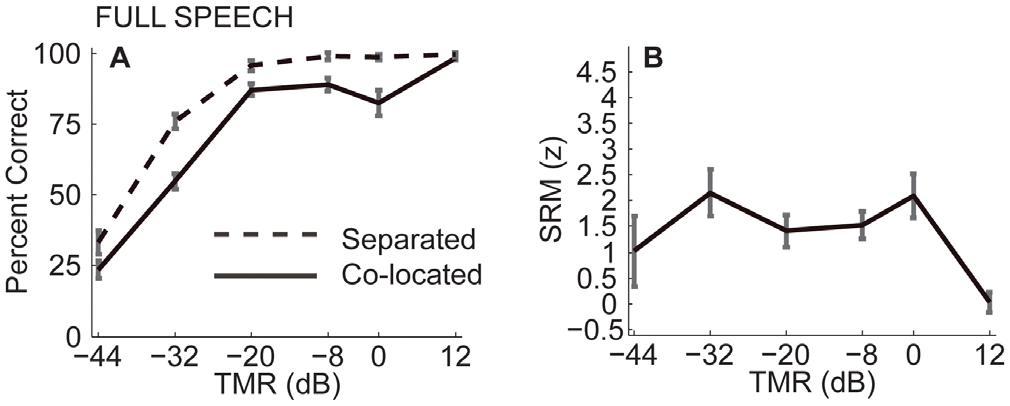
Performance from Experiment 1 with unprocessed speech as a function of target to masker broadband energy ratio (TMR). Error bars show estimated 95% confidence intervals after correcting for between-listener variance. A) Percent correct for spatially separated sources (dashed line) and co-located sources (solid line). Performance was better in the spatially separated than in the co-located configuration, a phenomenon referred to as spatial release from masking. B) Spatial release from masking (SRM), i.e., the difference between the dashed and solid lines in panel A), expressed in z-units (see text for details on Data Analysis).

Figure 3A shows percent correct performance as a function of TMR, for the co-located and separated configurations. Performance was similar across listeners. Therefore, only the mean performance across listeners is shown. Error bars show the estimated 95% confidence intervals around the mean. To that end, here and throughout the study, the standard error of the mean across listeners was computed, the between-listeners variance was removed, and estimates were calculated as t_alpha_ times this corrected standard error, where t_alpha_ is the t-value corresponding to alpha = 5% confidence level (cf., [39]). Performance in the co-located configuration, where both sources were at 0°, was consistently worse than performance in the separated configuration, where the target came from the side (solid line falls below dashed line). Repeated measures ANOVA on the logit-transformed percent correct scores found significant main effects of TMR [F(5,25) = 494.1, η_p_^2^ = 0.99, ω_p_^2^ = 0.99, p<0.001] and spatial configuration [F(1,5) = 815.3, p<0.001, η_p_^2^ = 0.99, ω_p_^2^ = 0.99]. The interaction between TMR and spatial configuration was significant [F(5,25) = 34.1, p<0.001, η_p_^2^ = 0.87, ω_p_^2^ = 0.87]. Post-hoc LSD testing found significant differences between pairwise comparisons of spatial configurations for all tested TMRs except +12 dB TMR (where p = 0.018, <0.001, 0.001, <0.001, <0.001, 0.78 for −44, −32, −20, −8, 0, and 12 dB TMR). At 12 dB TMR, in the spatially separated configuration, performance was limited by ceiling, unlike in the co-located configuration.

Moreover, in the co-located configuration, performance did not increase monotonically. Instead, at 0 dB TMR performance was worse than, or at best equal to, that at −8 dB TMR. This is consistent with previous results showing that in a two-source mixture, level differences between competing sources can help listeners attend to the target sound ([23], [38], [5]).

Figure 3B shows the amount of SRM. At +12 dB TMR, where performance in both spatial configurations was close to ceiling (dashed line near 100% correct), SRM was smallest overall. At −8 and 0 dB TMR, performance in the spatially separated condition was close to ceiling, perhaps causing underestimation of SRM at these TMRs. Except for +12 dB TMR, the amount of SRM did not vary much with TMR. Across-subject across-TMR average SRM equaled 1.9 z-units, with a 0.2 z-unit SE, translating to approximately 11%.

### Experiment 2

**Figure 4:**
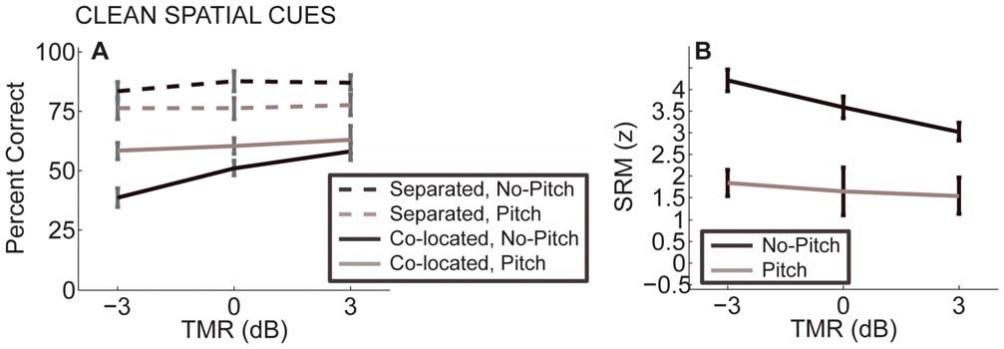
Performance from Experiment 2 with chess-vocoded speech with clean ITD cues as a function of TMR. Error bars show 95% confidence intervals. A) Percent correct for spatially separated sources (dashed line) and co-located sources (solid line). B) SRM. Performance was consistently better in the spatially separated than in the co-located configurations. SRM was greater for the *No-Pitch* condition than for the *Pitch* condition.

Figure 4A shows performance in the spatially co-located and separated conditions (solid and dashed lines, respectively) when target and masker spectra were in *Pitch* or *No-Pitch* configuration (black and grey lines, respectively). Similar to Experiment 1, here, in all stimulus conditions, performance was better in the spatially separated than in the co-located configurations (dashed lines are above solid lines). Moreover, in the co-located configurations, performance in the *Pitch* condition was better than in the *No-Pitch* condition, suggesting that listeners were able to use place pitch cues to select the target message. In the spatially separated configuration, however, performance was worse for the *Pitch* than for the *No-Pitch* condition, indicating that spatial cues were overall less useful when listeners could use pitch cues. Previous studies show that spatial cues can be less helpful and spatial release from masking smaller when spatial cues are redundant with level and/or pitch cues ([10], [38], [40]).

Statistical analysis supports these interpretations. Repeated measures ANOVA found significant main effects of TMR, and spatial configuration [F(2,10) = 20.8,p<0.001, η_p_^2^ = 0.81, ω_p_^2^ = 0.80, F(1,5) = 132.7,p<0.001 η_p_^2^ = 0.96, ω_p_^2^ = 0.96]. The main factor of spectrotemporal condition was not significant [F(1,5) = 2.0, p  = 0.216, η_p_^2^ = 0.29, ω_p_^2^ = 0.17]. The interaction between spatial configuration and spectrotemporal condition, however, was significant [F(1,5) = 327.4, p<0.001, η_p_^2^ = 0.99, ω_p_^2^ = 0.99]. For both *Pitch* and *No-Pitch* spectrotemporal conditions, post-hoc LSD test revealed significant differences for pairwise comparisons between the two spatial configurations [p = 0.001 and p<0.001 for *Pitch* and *No-Pitch*]. This is consistent with worse performance in the co-located *No-Pitch* than in the co-located *Pitch* condition. In contrast, in the spatially separated conditions, *No-Pitch* performance was better than *Pitch* performance.

Figure 4B shows SRM in the *No-Pitch* and *Pitch* configurations (denoted by black and grey lines, respectively). SRM was consistently greater for the *No-Pitch* condition, where place pitch cues were absent, than for the *Pitch* condition, where the lower bands could convey place pitch as a cue to select the target from the mixture. Repeated measures ANOVA of SRM found a significant main effect of spectral condition and of TMR [F(1,5) = 185.0, p<0.001, η_p_^2^ = 0.97, ω_p_^2^ = 0.97 and F(2,10) = 6.7, p = 0.014, η_p_^2^ = 0.57, ω_p_^2^ = 0.53]. The interaction between spectral condition and TMR was significant [F(2,10) = 6.8, p = 0.014, η_p_^2^ = 0.58, ω_p_^2^ = 0.54]. Pairwise comparison with post-hoc LSD test found significant differences between *Pitch* and *No-Pitch* at all three TMRs (p<0.001 in all cases). In the *Pitch* condition, SRMs did not differ significantly across the three TMRs (p = 0.529, 0.798, 0.168 for SRMs at −3 dB versus 0 dB, 0 dB versus 3 dB, and −3 dB versus 3 dB). In contrast, SRM decreased with increasing TMR in the *No-Pitch* condition (p = 0.017, 0.014, <0.001 for SRMs at −3 dB versus 0 dB, 0 dB versus 3 dB, and −3 dB versus 3 dB).

### Experiment 3

**Figure 5:**
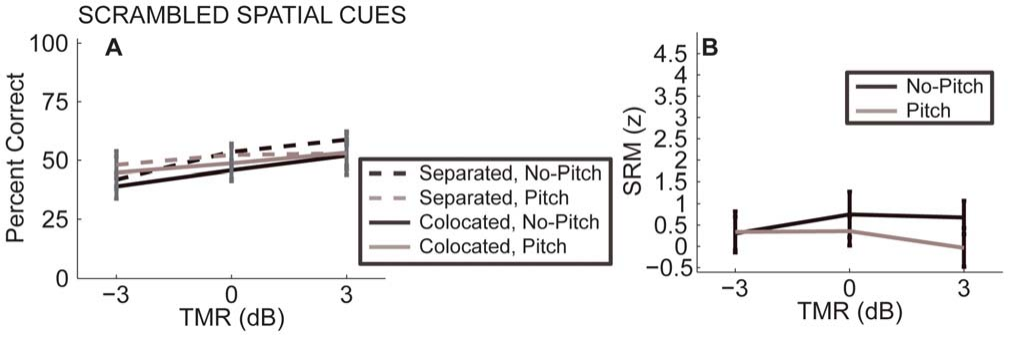
Performance from Experiment 3 with chess-vocoded speech with scrambled ITD cues as a function of TMR. Error bars show 95% confidence intervals. A) Percent correct for spatially separated sources (dashed line) and co-located sources (solid line). B) SRM. Dashed and solid lines nearly overlap, and SRM is close to zero for both the *No-Pitch* and the *Pitch* condition.

Figure 5A shows performance in co-located and separated configurations, with *Pitch* and *No-Pitch* spectral conditions (solid and dashed lines denote co-located and separated configurations; grey and black lines show *Pitch* and *No-Pitch* conditions). Overall performance was slightly better in the spatially separated compared to the co-located configurations (dashed lines are above solid lines), and slightly better for *Pitch* than for *No-Pitch* conditions (black solid and dashed lines fall below grey solid and dashed lines). However, these differences were not statistically significant. Repeated measures ANOVA found a main effect of TMR [F(2,10) = 31.6, p<0.001, η_p_^2^ = 0.86, ω_p_^2^ = 0.86], but did not turn up significant effects of spatial configuration or spectrotemporal condition [F(1,5) = 5.6, p = 0.06, η_p_^2^ = 0.53, ω_p_^2^ = 0.48, F(1,5) = 0.3, p = 0.6, η_p_^2^ = 0.06, ω_p_^2^ = 0.00]. The effect of spatial configuration was marginally non-significant, indicating that there was a trend to utilize spatial information even with these highly degraded spatial cues.

Figure 5 B shows SRM. SRM was generally close to zero. Repeated measures ANOVA found no significant effects of spectral condition or TMR [F(1,5) = 1.329, p = 0.30, η_p_^2^ = 0.21, ω_p_^2^ = 0.06 F(2,10) = 1.304, p = 0.31, η_p_^2^ = 0.21, ω_p_^2^ = 0.06].

### The Role of Clean ITDs

**Figure 6:**
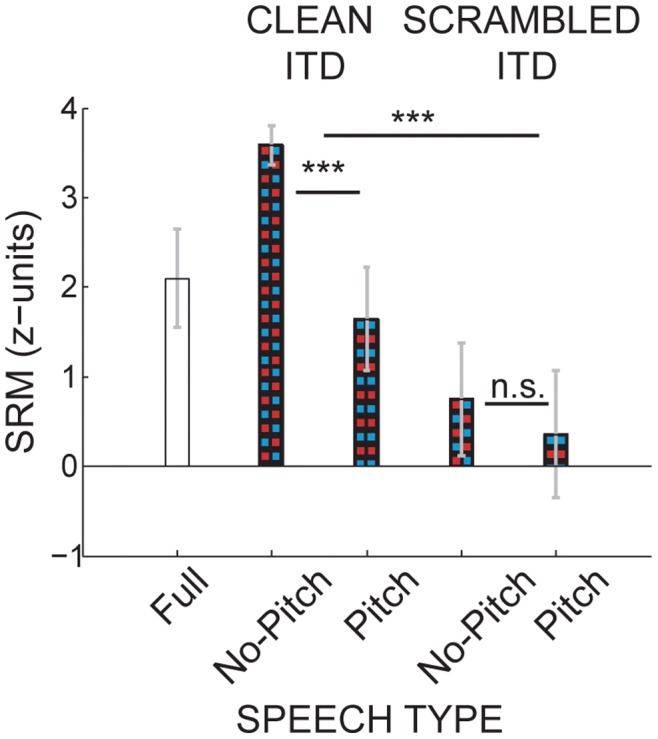
Comparison of SRM across experiments. SRM at 0 dB TMR (data replotted from Figs. 3, 4, 5). SRM is greater in the clean-ITD conditions compared to the scrambled-ITD conditions. Across the two clean-ITD cases, more spatial release occurred when place-pitch cues were absent than when they were present.

Figure 6 plots SRM scores from all three experiments. To eliminate the potentially confounding effects of level cues, only results at 0 dB TMR were included. Comparing results across Experiments 2 and 3, SRMs were greater for clean than for scrambled ITD cues. Moreover, they were greater in the *No-Pitch* than in the *Pitch* condition. Indeed, a mixed-model repeated measures ANOVA with between-subjects factor of spatial cue fidelity found a significant difference between SRM with clean and scrambled ITDs [F(1,10) = 43.0, p<0.001, η^2^ = 0.19, ω_p_^2^ = 0.73]. Furthermore, the effect of spectrotemporal condition was significant [F(1,10) = 30.47, p<0.001, η^2^ = 0.52, ω_p_^2^ = 0.79]. The interaction between pitch condition and spatial cue case was significant [F(1,10) = 18.13, p  = 0.001, η^2^ = 0.11, ω_p_^2^ = 0.61]. Post-hoc LSD pairwise comparison showed that in the clean-ITD cases, SRMs differed significantly between *Pitch* and *No-Pitch* conditions (p<0.001). However, in the scrambled-ITD cases, SRMs were not statistically different between *Pitch* and *No-Pitch* (p = 0.324).

## Discussion

When listening for a target in the presence of a masking talker, for normal-hearing listeners, spatially selective attention can help identify the target speech (e.g., [41], [8]). While it is unclear whether cochlear implant listeners can utilize spatial attention, preliminary evidence suggests that their ability to use spatial cues for source identification is strongly limited ([24]). Because of technical limitations in bilateral cochlear implant processors, the best opportunity for restoring attention driven SRM in cochlear implants could lie in ILDs. Here, three experiments examined whether, in a simulation of cochlear implant listening, it is possible to obtain SRM with ILD cues even when ITD cues are scrambled across time and frequency. Long-term average acoustic ILDs were similar across experiments, but ITD fidelity varied. We were specifically interested in performance at very small TMRs, where level cues do not help much with selecting a target ([5],[23], [38]). However, small ranges of tested TMRs strongly limit the precision of estimated slopes and midpoints of the underlying psychometric functions and thus the precision of the resulting estimated SRMs in dB. Our solution was to compare the magnitude of the underlying spatial release in units that have been transformed to soften the effects of floor and ceiling performance and normalized by the underlying response variance to allow comparison across performance scores of different observed means and variances. Expressed this way, we could directly contrast SRM at 0 dB TMR across different performance levels.

There was little if any spatial release in the scrambled-ITD conditions of Experiment 3, and SRM was significantly smaller than SRM in the clear-ITD conditions of Experiment 2. In fact, SRM was overall greatest in the *No-Pitch* clean-ITD cases of Experiment 2, even when compared to unprocessed speech with high-fidelity spatial acoustic cues. These results demonstrate that faithful ITD cues are necessary to restore SRM.

Moreover, in both Experiments 2 and 3, SRM was smaller in the *Pitch* than in the *No-Pitch* conditions. This highlights the potential usefulness of spatial cues when other segregation cues (such as pitch, onset cues or level) are impoverished. In general, over short time spans of several milliseconds, such as the duration of the “chess board” glimpses in this study, spatial cues contribute little weight to how a listener segregates target elements from an acoustic mixture ([21], [42], [43]). However, over longer time spans, spatial cues are helpful in allowing the listener to select a target voice from a mixture of competing streams ([41], [5]). Together, the current results buttress and extend previous findings that SRM tends to be smaller when other segregation cues are also present compared to when only spatial cues are available ([5], [44], [40]).

In one previous study, when ITDs were zero and ILDs were held at a fixed value, speech reception thresholds for normal hearing listeners identifying speech masked by three speech interferers were similar to those expected from better-ear listening ([45]). If spatial attention had contributed to performance in that previous task, SRM should have exceeded that of better-ear listening, but that was not observed. Our approach differs from this previous study in that here, ITD was scrambled to simulate the information available for cochlear implant listeners. The resulting percept is spatially wider than for zero-ITD stimuli, but similar to the previous study in that it generates an acoustic image off the midline when ILDs are present. Moreover, unlike in the study by Culling et al., here, we presented target and masker with strongly reduced mutual energetic overlap. Therefore, we did not expect that the audibility of the target glimpses would strongly affect performance ([4], [5]). Rather, we expected that performance would be limited by how effectively listeners could deploy attention to identify the correct keywords. As a result, improving the theoretically infinite target to masker energy ratio in each target glimpse through the head shadow should not have improved performance.

Supporting this notion, in the scrambled cue cases of Experiment 3, listeners did not appear to benefit from better ear acoustic advantage. If the acoustic head shadow had improved performance, based on the analysis shown in Fig. 2F, where ILDs are at least 5 dB in both *Pitch* and *No-Pitch* cases in the scrambled-ITD cases, the psychometric functions should have at least been horizontally shifted relatively to each other by 5 dB. Even though the shallow slope of these functions makes it difficult to gauge the horizontal shift, this was clearly not observed for the Pitch case, where the psychometric functions are nearly identical across the two spatial configurations (compare dashed and solid grey lines in Fig. 5).

One possible explanation for why ILD cues alone do not allow for spatial release from masking when ITDs are scrambled is that in the spatially separated configuration, the perceived location of the target source may not have been distinct enough from that of the masker. In general, when ITD and ILD provide conflicting information about source direction, the information in the ITDs dominates the overall perceived location, both in anechoic conditions ([46]) and when reverberant energy degrades binaural cues ([33]). However, even in the scrambled ITD conditions, all listeners could at the beginning of each session reliably identify source direction with extremely high accuracy. Therefore, perceived location uncertainty due to ITD jitter is unlikely to account for the greatly reduced spatial release in the scrambled-ITD conditions.

The current results are in agreement with findings from hearing impaired listeners. Similar to cochlear implant listeners, hearing impaired listeners receive much less SRM than NH listeners, even after accounting for reduced audibility ([47]). In part, this lack of SRM may be due to the fact that hearing aids can alter ITDs received by the listener compared to acoustic ITDs ([47]), although alternative explanations, including impoverished temporal fine structure cues and impoverished spectral cues in hearing impaired listeners, have also been proposed ([48]).

Together, findings from the current study show that faithful long-term ILD cues by themselves do not suffice for directing spatial attention to a perceptually similar talker in a two-talker setting. Restoration of ITD cues should help cochlear implant listeners, and perhaps hearing impaired listeners, above and beyond enabling them to localize sounds, by providing them with an opportunity to utilize spatial attention.

### Conclusions

1. Spatial cues are more helpful and spatial release from masking is greater when place pitch cues are absent compared to when place pitch is conveyed.
2. When vocoded speech is processed to simulate loss of ITD cues while maintaining across-time average ILD cues, spatial release from masking reduces dramatically compared to when both ITD and ILD cues are maintained.
3. Results suggest that CI listeners can regain access to spatial release from masking if appropriate ITD cues are restored. Restored spatial release would greatly improve speech intelligibility in the presence of competing sources.
